# Healthy Bread Initiative: Methods, Findings, and Theories—Isfahan Healthy Heart Program

**DOI:** 10.3329/jhpn.v31i1.14748

**Published:** 2013-03

**Authors:** Mohammad Talaei, Noushin Mohammadifard, Mohammad-Reza Khaje, Nizal Sarrafzadegan, Firoozeh Sajjadi, Hasan Alikhasi, Maryam Maghroun, Farhad Iraji, Shahram Ehteshami

**Affiliations:** ^1^Cardiovascular Research Center, Isfahan Cardiovascular Research Institute, Isfahan University of Medical Sciences, Isfahan, Iran;; ^2^Public Health, Epidemiology & Biostatistics, University of Birmingham, U.K.;; ^3^Faculty of Management and Medical Information, Isfahan University of Medical Sciences, Isfahan, Iran;; ^4^Isfahan Health Center, Isfahan University of Medical Sciences, Isfahan, Iran

**Keywords:** Bread, Community trial, Health promotion, Nutrition, Iran

## Abstract

The scientific evidences show that the content, baking methods, and types of bread can make health impacts. Bread, as a major part of Iranian diet, demonstrates a significant potential to be targeted as health promotion subject. Healthy Food for Healthy Communities (HFHC) was a project of Isfahan Healthy Heart Program (IHHP), consisting of a wide variety of strategies, like Healthy Bread (HB) Initiative. The HB Initiative was designed to improve the behaviour of both producers and consumers, mainly aiming at making high-fibre, low-salt bread, eliminating the use of baking soda, providing enough rest time for dough before baking (at least one hour), and enough baking time (at least one minute in oven). A workshop was held for volunteer bakers, and a baker-to-baker training protocol under direct supervision was designed for future volunteers. Cereal Organization was persuaded to provide less refined flour that contained more bran. Health messages in support of new breads were disseminated by media and at bakeries by health professionals. Evaluation of the HB Initiative was done using before-after assessments and population surveys. While HB was baked in 1 (0.01%) bakery at baseline, 402 (41%) bakeries in the intervention area joined the HB Initiative in 2009. Soda was completely eliminated and fibre significantly increased from 4±0.4 g% before study to 12±0.6 g% after the intervention (p<0.001). The preparation and baking times remarkably increased. Wastage of bread decreased from 13±1.8 g% to 2±0.5 g% and was expressed as the most important advantage of this initiative by consumers. People who lived in Isfahan city consumed whole bread 6 times more than those who lived in reference area Arak (p<0.001). The HB Initiative managed to add new breads as a healthy choice that were compatible with local dishes and made a model to solve the long-standing problems of bread. It used various health promotion approaches but was best consistent with Beattie's model.

## INTRODUCTION

Bread has been considered “staff of life” since the beginning of recorded time. A characteristic of the Iranian diet is the dependence on bread and rice as the major energy sources (1). Several epidemiological studies have shown that intake of whole-grain products is associated with reduced risk of type 2 diabetes, hypercholesterolaemia, metabolic syndrome, and coronary heart disease as well as cardiovascular mortality (2-6). Empirical evidence shows that consumption of whole-wheat meals, like whole-grain bread, compared to white bread, even for 3 or 4 weeks, reduces total serum cholesterol level (2), LDL cholesterol, non-HDL cholesterol, triglyceride (3), body fat, and abdominal obesity (4,5). It might be because of the high fibre content in this kind of bread. Furthermore, it improved the bioavailability of vitamins and minerals, such as varieties of vitamin B, calcium, and magnesium (6). Thus, it has greater diet quality and is attributed to lower risk of cardiovascular disease (CVD). These facts make bread a relevant subject for health promotion.

The Iranians are traditionally accustomed to fresh hot bread; so, bakers have to do their business only in special short periods of day before breakfast, lunch, and dinner. Consequently, bakeries encounter queue of consumers during the mentioned times while they are idle for the rest of the day. Therefore, there has been little room for industrial bread business compared to small-scale local bakeries. There are technical problems in producing this thin bread as well as in keeping it. On the other hand, modernization made it possible to refine grains much more than before and as the fallacy of “appeal to novelty” usually works, the white flatbread came in vogue. Under these circumstances, bakers tend to add sodium bicarbonate, also known as baking soda (NaHCO_3_) to dough as a substitute for natural fermentation process. It releases carbon dioxide (CO_2_) at baking temperatures (>60 °C) and helps dough rise. This innovation eliminates the need for dough rest time and reduces work pressure on bakers but is not healthy and decreases the keeping quality of bread, particularly out of fridge (at room temperature) to less than one day. Several attempts were made to deal with the situation but almost all were not succeeded, and bread production and consumption pattern remained nearly unchanged.

As a public-health response to the high prevalence of CVDs in Iran, a six-year action-oriented comprehensive and integrated community-based intervention titled “Isfahan Healthy Heart Program (IHHP)” was designed and launched in 2001. The long-term objectives of IHHP were to decrease the incidence of non-communicable diseases (NCDs), including CVD, diabetes, hypertension, cancers as well as to decrease disability and mortality associated with NCDs. The short-term objectives were to improve knowledge and awareness about the causes and consequences of NCDs in the general population and among health professionals, to improve individual skills to control risk factors and promote healthy behaviours. The IHHP objectives were described in details elsewhere. Healthy Food for Healthy Communities (HFHC) was a component of IHHP project, consisting of a wide variety of strategies, including initiatives regarding oil consumption and labelling as well as intake of healthy bread (7,8,9,10).

The goal of the Healthy Bread (HB) Initiative was to introduce this product to an urban community and increase its consumption. This report aimed to describe the experience of implementing the HB Initiative and its supporting theories, to criticize it from health promotion perspective, to explain supportive activities, and to report the project's outcomes and shortcomings at the community level as well as individual level.

## MATERIALS AND METHODS

The IHHP was launched in two interventional areas (Isfahan and the neighbouring Najafabad city) and one reference area Arak—all located in central part of Iran. After a baseline survey in 2001, a five-year interventional programme was started in August 2001 in both urban and rural areas of Isfahan and Najafabad. Priorities and needs were assessed, and objectives were constructed based on baseline findings. The existing human and economic resources were used, and the plans and strategies were set emphasizing tobacco control, healthy diet, physical activity, and stress management based on different target groups. Interventions continued until 2006. The IHHP was confirmed via external evaluation (11).

The IHHP strategies had integrated activities covering different fields, including health promotion, disease prevention, healthcare, and rehabilitation. The programme comprised 10 distinct projects, each targeted to different fields and groups, one of which was HFHC (7,8). In terms of healthier nutrition, main emphasis was put on activities toward changing the dietary environment of the population. Several activities with a wide range of stakeholders were undertaken to improve the foods such as those served in restaurants and fast food shops and also produced in bakeries and food industries (10), one of which was the HB Initiative.

### Recipe development

From nutritional perspective, good bread has a range of standards from wheat cultivation to storage methods. In the HB Initiative, it was mainly intended to make high-fibre, low-salt bread, eliminating the use of baking soda, providing enough rest time for dough before baking (at least one hour) as well as enough baking time (at least one minute in oven). In order to fit all these criteria, some new kinds of bread had been invented. They were called ‘whole bread’, ‘whole-grain bread’, or ‘semi-voluminous bread’. The term ‘semi-voluminous’ means that the new bread was something between ordinary flat thin bread and industrial breads, like baguette, which the Iranians officially call ‘voluminous’. No new equipment was necessary for providing new bread.

### Implementation of HB Initiative

Before the beginning of the programme (2001), one bakery provided healthy bread and was interested in its promotion. The owner had a Masters degree in Education and was linked with a university department of nutrition with the purpose of training the students of nutritional science. His ancestors were also bakers. He was selected an agent for the HB Initiative. At first, 32 volunteer bakers were asked to attend a workshop that was led by nutrition professionals and the agent. The main theme of the workshop was the recipes of new healthy breads (including oat and maize) and the related techniques. Regarding new volunteers, the participants of that workshop were made local trainers in their corresponding sectors of the city. These training courses (baker-to-baker) were held under direct supervision of the HB agent.

**Figure 1.**
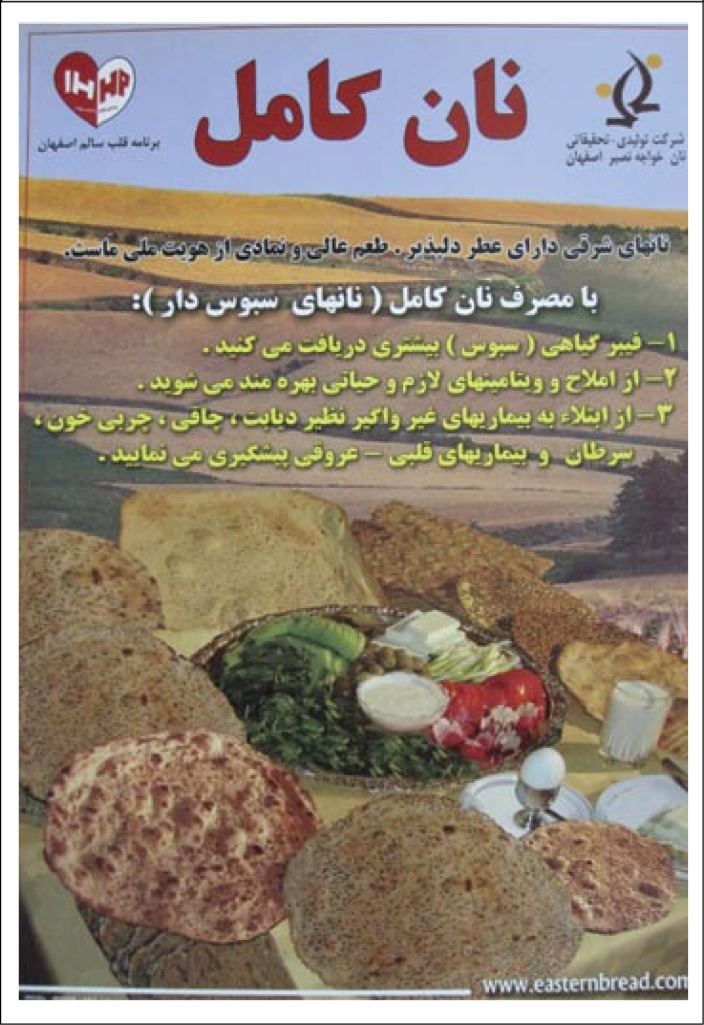
A poster of Healthy Bread Initiative

The IHHP leaders and the HFHC staff supported the new bread in its health value through leaflets, posters, and disseminating the issue of healthy bread in the media, alone or with other health issues. For instance, the poster of HB stated that people could prevent diabetes, obesity, high blood cholesterol, cancer, and cardiovascular diseases by consuming HBs (Figure 1). It was also stated on the poster that these kinds of bread could provide better supply of vitamins and minerals, and the importance of the higher quantities of plant fibres (bran) in HB was stressed. Moreover, the IHHP arm was clearly observable at the top of poster. Since this arm was used in several different projects, it was familiar to the people. The posters were put at bakeries that produced HB, and the leaflets were made available there. In TV shows, radio programmes, and newspaper articles, the advantages of HB and disadvantages of white flat bread were explained by professionals (nutritionists, cardiologists, etc.). The main goal was to change the wrong belief of the superiority of white bread compared to dark bread.

In the early years of the project, bran was separately added to wheat flour by bakers of HB. When the business of new bread grew and remarkable demands were created, the negotiations were opened with Cereal Organization to provide a kind of flour that was less refined. It finally caused this type of flour to become available.

The used flour in Isfahan province was completely provided by the Isfahan Grain Office. The HFHC collaborated with Isfahan Grain Office and its Quality Control section to improve the fibre content of the flour. In addition, after 3 years when HB had been introduced to the community, Isfahan Health Center was persuaded to issue a directive to forbid all bakers (including non-volunteers) from using soda (replacement by yeast) and decreasing amount of salt in bread. Afterwards, this directive was adopted at the national level by the Iranian Ministry of Health (12).

### Evaluation

Process evaluation of HB production was carried out using a 6-year before-after design with special checklists, which was completed by health professionals during their bimonthly visit to bakeries. Process evaluations of the whole IHHP interventions (13) as well as nutritional interventions, including the HB Initiative, were previously published (14). The information about wastage of wheat, flour, and bread was collected from the annual assessments by the Grain Office.

The opinion of people about the quality and wastage of HB and their satisfaction were assessed at the end of the study. A questionnaire was designed, and the validity of its content was confirmed by experts. Respondents were asked about three different aspects of the quality of bread, i.e. taste, brittleness, and persistence (durability). Those who reported better quality for at least 2 dimensions were assumed to believe that HB had overall better quality. The reliability of the questionnaire was assessed using a test-retest study among 20 adults with a two-week interval. Correlation coefficients of items ranged from 0.70 to 0.82. Twenty bakeries were randomly selected and, in each one, 2-3 clients were recruited, using convenient sampling method. In general, 45 subjects were interviewed.

The proportion of whole bread consumption in Isfahan city and Arak city were extracted from the IHHP database. The IHHP was evaluated based on annual surveys from 2001 to 2006, using cluster random-sampling method (independent samples) in Isfahan and Najafabad as intervention areas and Arak as the reference area. HB was not introduced in Najafabad region and rural area of Isfahan. Therefore, data on participants in Isfahan city were only compared with that from Arak city. Data from Najafabad city were excluded to avoid contamination by another intervention on salt reduction. Information on bread consumption was collected in 2004 and 2007 asking about daily use of all available types of bread based on servings, using a semi-quantitative food frequency questionnaire (n=9,559). Dietary behaviours were assessed with a validated 48-item food frequency questionnaire (FFQ) (15). For a random subsample (n=457) of the IHHP survey in 2007, a single 24-hour recall questionnaire was completed. Data from 24-hour recall questionnaire were computed by the Iranian Food Consumption Program (IFCP) designed by ICRC (16), based on Iranian Food Composition Table (17). Percentage of daily calories from these breads was derived from these data. Total fibre intake was calculated as the whole consumed fibres divided by energy expenditure multiplied to 1,000 (g fibre intake per 1,000 kcal).

### Statistical analysis

The data were entered into EPI Info and analyzed by SPSS (version 15). Logistic regression analysis was employed using whole bread consumption as dependent variable and place of residence, survey times, age, and sex as independent variables. The interaction of place of residence and survey times were also included in the model. Due to the severe skewness of bread intake in grammes, the numerical data were reported as median (interquartile range), and Mann-Whitney U-test was used for pairwise comparisons. Other numerical values were presented as mean±standard deviation. Paired *t*-test was used for comparing the mean level of baking time, dough-preparation time, soda content, and bread wastage before and after the intervention. The HB bakeries at baseline were compared with the frequency after implementing the HB project, using McNamar test; p values of less than 0.05 were considered statistically significant.

## RESULTS

The mean age of participants in the IHHP surveys was 41.1±16.6 years in Isfahan city and 40.7±16.2 years in Arak city (p=0.223). Among 9,559 respondents, 4,811 (50.3%) subjects were female, and there was no significant difference between the two areas (p=0.224).

**Figure 2.**
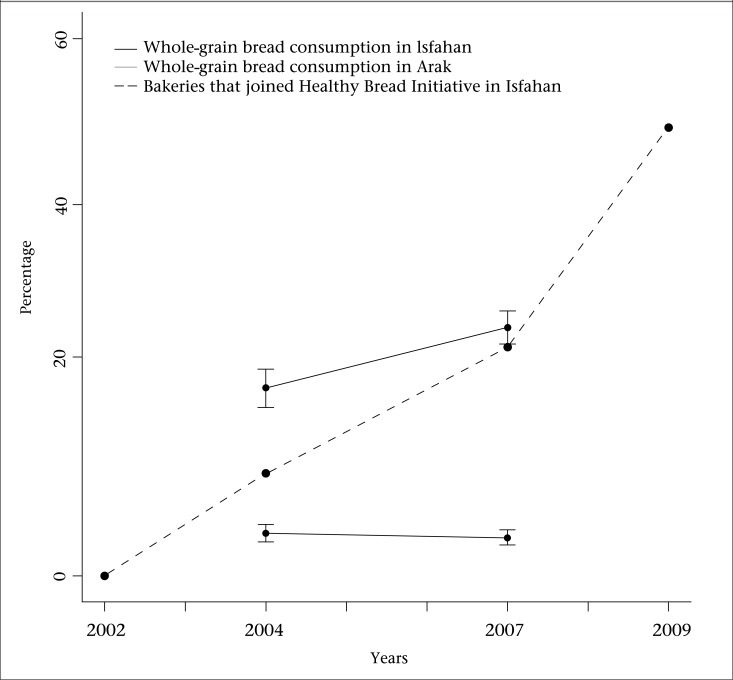
Consumption of whole-grain bread by population in the intervention and reference areas and the percentage of bakeries that joined Healthy Bread Initiative

Final evaluation showed that, in the intervention area, number of bakeries that produced HB increased from 1 (0.01%) at baseline in 2001 to 402 (41%) in final evaluation in 2009 (Figure 2). As a consequence, people who lived in Isfahan city consumed HB (whole bread) 6 times more than those who lived in the reference area in 2007 (interim evaluation, p<0.001). Accordingly, living in Isfahan remarkably increased the odds of daily intake of this bread (OR 5.1, 95% CI 3.9-6.6, p<0.001). In comparison with 2004, the possibility of starting consumption of whole-grain bread in Isfahan was 56% more than in Arak (OR for interaction 1.56, 95% CI 1.1-2.1, p=0.009). This association was not influenced by sex (p=0.764) and age (p=0.379). In 2004, the median amount of whole bread consumption was 50 (50) g and 25 (100) g in Isfahan and Arak respectively. It increased to 125 (100) g in Isfahan and 50 (43) g in Arak in 2007 (p< 0.001 and p=0.007).

HBs were, on average, 71% more expensive (3500 vs 2500 IRR/kg equivalent to US$ $0.35 vs $0.25/kg). The nutrition assessment that was done on a subsample of participants (n=457) in the final IHHP survey showed that 23.8% of daily energy was derived from HB in Isfahan in 2007. Fibre intake from bread was significantly higher in Isfahan (6.0±5.9 g/1,000 kcal) compared to the reference area Arak (5.3±4.3 g/1,000 kcal, p=0.042). However, total fibre intake was not significantly different in the intervention areas (9.8±4.1 g/1,000 kcal) compared to the reference area (9.7±3.6 g/1,000 kcal, p=0.671).

At the time when the report was prepared, the degree of refinement in 55% of flour production was 88% (12% of bran was extracted), and the rest of it (45%) was 93% refined (7% extracted). In addition, 2 of 5 units of the county's central mill were dedicated to producing whole-wheat flour rather than completely-refined flour. It increased the availability of whole-wheat flour to bakeries. Mean of soda and fibre contents as well as dough preparation and baking times are presented in the table.

**T1able.** Bread characteristics before (2001) and after (2007) Isfahan Healthy Heart Program

Characteristics	Before	After	p value
	Mean±SD	Mean±SD	
Soda (g%)	0.13±0.06	0	<0.001
Fibre (g%)	4±0.4	12 ± 0.6	<0.001
Wastage (g%)	13±1.8	2 ± 0.5	<0.001
Preparation time (hour)	0.3±0.04	2 ± 0.18	<0.001
Baking time (minute)	13±1.8	2 ± 0.5	<0.001
Healthy bread bakery n (%)	1 (0.01)	402 (41)	<0.001

SD=Standard deviation

Among 45 participants in the population opinion survey, 3 (6.6%) always used HB, 16 (35.5%) mostly, and 19 (42.2%) occasionally consumed it but 7 (15.5%) subjects stated that they never used HB. Thirty-four (75.5%) respondents reported that HB had generally better quality while 7 (15.5%) subjects believed it was worse, and 4 (8.8%) participants had no comments. The most important advantage of the new bread was expressed by consumers to be fewer bread wastes, and 34 (75%) subjects reported that HB had less waste but 7 (15.5%) respondents stated that it had more wastes; 3 (6.6%) subjects believed in similar wastes, and one (2.2%) had no comments.

The manageable wastage of bread is inherent in baking and preparation techniques rather than people's consumption or keeping methods. According to the report of Isfahan Grain Office, the manageable wastage of bread, which constitutes 11% of bread production, was decreased to 5% at the end of the study.

## DISCUSSION

This study introduced a healthy product, namely HB, to the community and managed to distribute it to some extent. Considering complete fermentation process in HB, it needed less salt and no added sodium bicarbonate. Compared to the ordinary breads in the region, HB consisted of whole grain and contained more bran. The new product was called ‘healthy’ due to the less salt, no sodium bicarbonate, and more bran in its composition. American Heart Association and American Diabetes Association stressed the importance of grain intake and recommended to take at least half of grain from whole-grain breads (18,19). Based on 24-hour urine analysis, the salt intake of people in Isfahan was 8.2 g/day in 1999-2000; it increased to 12.5 g/day in 2001-2002 but decreased to 10.6 g/day in 2007 (20). As bread constitutes an important part of daily diet of people in the region, the reduction of bread's salt may, to some extent, result in such reduction. On the other hand, absence of sodium bicarbonate as well as complete fermentation process (due to longer preparation time) and greater thickness resulted in more persistence that, in turn, caused less bread wastes. Although inhabitants of Isfahan had more fibre intake from bread, it failed to increase total fibre intake compared to the reference area. This could be attributed to the higher fibre intake from other sources in the reference area.

### Underlying theories

The HB Initiative components were partly in line with three approaches of Ewles & Simnett model as a normative one (21). It was a clear attempt to persuade community members with messages that dark bread is better and to encourage them to adopt healthy bread diet through provision of information, using an expert-led, top-down approach (behaviour change approach). On the other hand, as the main efforts were put into providing healthy choice, it can be considered as an attempt to change environment. Nevertheless, social change approach has been summed up as “to make the healthy choice, the easier choice” (22). Consequently, for lack of any plan regarding cost of the new breads, HB does not thoroughly comply with social change. Educational approach was also used because information in favour of bran and against salt was broadly disseminated in the society without particular direction.

Considering four paradigms of Beattie's model (22) of health promotion, including health persuasion by healthcare professionals, personal counselling, legislative action, and community development, only personal counselling was not definitely in agreement with this initiative. Since the target of messages via mass media was individuals and these were clearly prescriptive, health persuasion is applicable to the HB Initiative. In terms of bakers, mode of intervention were bottom-up and top-bottom, and focus was collective, including lobbying with organizations and bakers syndicate, action research, skills sharing, training (both nutritionist-to-baker and baker-to-baker), and group work. However, as bakers voluntarily participated, it had some aspects of negotiated mode (valuing autonomy) and might be better to place between legislative action and community development. Moreover, some parts of those activities, like banning baking soda and decreasing salt, had definite authoritative style and indicated legislative paradigm. Other dimensions, including physical, mental, spiritual and environmental factors that are essential for total health and well-being, were not included in Beattie's model (23); so, some aspects of HB could not be explained completely.

### Advantages and disadvantages

In line with multidisciplinary public-health concept, HB encompassed numerous approaches, and it made relative success. Previous attempt at local and national levels did not reach acceptable success level because these did not benefit in a holistic view. For example, enforcing legal restriction and bans could never solely eliminate undesirable methods and techniques of bakers as it had been futile before. Another example is industrial breads that could not substitute the ordinary ones. Method of baking is the consequence of many factors, including knowledge and attitude of consumers and bakers, people's taste, types and diversity of desired dishes, physical situation, and tradition. Since baking style had been developed during decades without taking all aspects into account, no real change could be made. Even in this initiative, there is some doubt toward the consequences of legislative approaches, especially in those bakers who did not join this programme. The critics may argue it is naive to expect that a directive by itself could have eliminated baking soda from ordinary breads when other factors were still persistent.

Apart from the need to work with bakers, selection of bakeries as health promotion setting to propagate health messages (via posters and leaflets) had the situational advantage. In Iran, people usually have to wait in queue to buy bread. Health messages, especially nutrition-related ones, could be better transmitted there rather than in pharmacies or a health centre that does not usually have very friendly atmosphere. However, these messages only targeted those who were interested in HB bakeries and the family members who were in charge of providing daily food, which can be a disadvantage. This gap is not necessarily filled by messages in mass media but using combination of different channels in itself was definitely a plus point, particularly when source of messages was a research centre or a nutritionist with improbable connection to the bakeries business.

The poster (Figure 1) had few words but impressive images of several different types of HBs. Those breads were laid around a tray full of vegetables and dairy products. It not only aroused curiosity of observers but also represented a nostalgic and traditional, simple, healthy and cheap meal. It had the potential to attract the attention of more people, including deprived groups, provided they come to HB bakeries. However, the formal scientific wording styles in both leaflets and the posters may fail to attract socially-deprived people. On the other hand, as HB was not voluminous, it looked like ordinary flat breads. Therefore, people did not see something unfamiliar on the poster. It was important because conventional local dishes and eating style of the Iranians (hand involvement) as well as people of many neighbouring countries in the Middle East are not compatible with voluminous breads, like baguette.

Preventive measures that bring much benefit to the population yet offers little to each participating individual. It is because the determinants of incidence at population level are not necessarily the same as the causes of disease in each individual. The failure to acknowledge this phenomenon more directly in health education material, which is called ‘prevention paradox’ can lead at best to greater mistrust among the general public toward the message, and at worst to their outright rejection (24). In fact, interventions such as HB only decrease the ‘risk’ of disease. This very subtle point makes remarkable difference in the way that it means to the community. It should be considered in community education and social marketing activities for products, like HB.

The HB Initiative benefited from introducing diversity of products to community, leading many people to welcome it. In addition, from five mentioned characteristics that have been known to be associated with successful adoption (25), HB only failed to fulfill “observability of results of adopting the innovation to others.”

One of the benefits of the new bread was low wastage. Bread wastage is not only uneconomical but also carries a religious stigma for people. The Government repeatedly expressed the interest in any solution to decrease wheat import. Consequently, HB had the potential to be presented beyond health issues through emphasizing on low wastage for both people and the Government, particularly Cereal Organization, which fulfills “the clarity of relative advantage.” The new breads did not need new setting or instrument, and bakers could lead training; this programme met both ‘simplicity’ and ‘flexibility’ characteristic of success as well as ‘reversibility’ and perceived low risk of adoption.

HB was compatible with cultural values of adopters but not completely with the community's prevailing socioeconomic status. It was owing to incomplete subsidies for whole-wheat flour while white flour received complete subsidy from government (beyond the local organizations). In fact, one of the most important shortcomings of this initiative was the higher cost for clients. Wheat was remarkably subsidized in Iran and was a cheap commodity. However, very deprived people were still vulnerable and probably could not afford HBs. As mentioned before, there is significant doubt in interpenetration of social marketing and educational activities in socially-deprived groups, which, alongside the cost issues, may have made this programme slightly widen poverty gap in health. In general, it seems that this initiative had been in complete disregard of deprivation issue.

HB was an innovation both as a new product for the community and as a new practice for bakers. Selection of a baker as agent was a strength, and his leading role as a model was pivotal to the success of HB. Apart from bakers’ goodwill, it should not be missed that producing something that a research centre undertakes the responsibility of its advertisement could be considered a motive from both economic standpoint and social prestige. HB established an innovative partnership and expanded it by adding Cereal Organization.

According to the diffusion of innovation theory, adopters are classified into innovators (2-3%), early adopters (10-15%), early majority (30-35%), late majority (30-35%), and laggards (10-20%) (25). In spite of all efforts and advantages, HB could not go beyond early majority and did not manage to thrive more after 6 years. There was no plan to speed up adoption of late majority and the laggards. However, the bakers joined increasingly in the HB Initiative during 2007-2009. The dynamics of social changes may have kept improving the penetration of HB market and have increased its usage. Perhaps, doing a randomized clinical trial in the future with this new bread could help encourage the ‘late majority’ and ‘laggards’ to adopt HB.

The fact that high price of the new bread is due to policy and is not inherent makes room to reverse the situation through negotiation and expanding partnership beyond the local level. This will make HB a complete social development element and might make a rapid increase in diffusion. Partnership has even more potential to develop HB. For instance, religious leaders are also interested in the reduction in bread wastage and have significant influence on low socioeconomic groups as well as bakers. Moreover, other businesses, like food shops, could be invited to join this initiative, perhaps by inventing different kinds of bread. Using bottom-up approaches, bakers syndicate could play a more important role to include bakers’ complaints and solutions as well as promote the concept “participate for the purpose of self-help” (26). NGOs could also be established to involve consumers.

### Conclusions

The HB Initiative managed to add new breads as healthy choices because these had no bicarbonate, less salt, and more bran. The initiative managed to promote its products that were compatible with local dishes and made a model to solve the long-standing problems of bread. It used health persuasion and legislative approaches to lead bakers to make new breads and to convince people about the superiority of dark breads. However, taking into account either advertisement or price; it overlooked the deprivation issues and the importance of providing healthy choices in a way that decreases inequality. There is much room in the HB Initiative for more innovation and development in terms of partnership and empowerment.

## ACKNOWLEDGEMENTS

This programme was conducted by the Isfahan Cardiovascular Research Center (ICRC)—a WHO collaborating centre—with the collaboration of Isfahan Provincial Health Office, both of which are affiliated with the Isfahan University of Medical Sciences. The programme was supported by a grant (No. 31309304) from the Iranian Budget and Planning Organization as well as the Deputy for Health of the Iranian Ministry of Health and Medical Education and the Iranian Heart Foundation. We are thankful to the team of ICRC, Isfahan Provincial Health Office, collaborators from Grain Office of Isfahan, and all bakers that joined the HB Initiative. The original draft was prepared for Health Promotion Module in the University of Birmingham; hence, our sincere thanks are extended to Dr. Robert Williams and the other lecturers for their invaluable comments.
